# Review of Audiovestibular Symptoms Following Exposure to Acoustic and Electromagnetic Energy Outside Conventional Human Hearing

**DOI:** 10.3389/fneur.2020.00234

**Published:** 2020-04-28

**Authors:** Rory J. Lubner, Neil S. Kondamuri, Renata M. Knoll, Bryan K. Ward, Philip D. Littlefield, Derek Rodgers, Kalil G. Abdullah, Aaron K. Remenschneider, Elliott D. Kozin

**Affiliations:** ^1^Warren Alpert Medical School of Brown University, Providence, RI, United States; ^2^Department of Otolaryngology, Harvard Medical School, Boston, MA, United States; ^3^Department of Otolaryngology, Massachusetts Eye and Ear Infirmary, Boston, MA, United States; ^4^Department of Otolaryngology, Johns Hopkins University School of Medicine, Baltimore, MD, United States; ^5^Walter Reed National Military Medical Center, Bethesda, MD, United States; ^6^Madigan Army Medical Center, Tacoma, WA, United States; ^7^Department of Neurosurgery, UT Southwestern Medical Center, Dallas, TX, United States; ^8^Department of Otolaryngology, University of Massachusetts Medical Center, Worcester, MA, United States

**Keywords:** audiovestibular disturbance, acoustic waves, electromagnetic energy exposure, infrasound, audible sound, ultrasound, radiofrequency

## Abstract

**Objective:** We aim to examine the existing literature on, and identify knowledge gaps in, the study of adverse animal and human audiovestibular effects from exposure to acoustic or electromagnetic waves that are outside of conventional human hearing.

**Design/Setting/Participants:** A review was performed, which included searches of relevant MeSH terms using PubMed, Embase, and Scopus. Primary outcomes included documented auditory and/or vestibular signs or symptoms in animals or humans exposed to infrasound, ultrasound, radiofrequency, and magnetic resonance imaging. The references of these articles were then reviewed in order to identify primary sources and literature not captured by electronic search databases.

**Results:** Infrasound and ultrasound acoustic waves have been described in the literature to result in audiovestibular symptomology following exposure. Technology emitting infrasound such as wind turbines and rocket engines have produced isolated reports of vestibular symptoms, including dizziness and nausea and auditory complaints, such as tinnitus following exposure. Occupational exposure to both low frequency and high frequency ultrasound has resulted in reports of wide-ranging audiovestibular symptoms, with less robust evidence of symptomology following modern-day exposure via new technology such as remote controls, automated door openers, and wireless phone chargers. Radiofrequency exposure has been linked to both auditory and vestibular dysfunction in animal models, with additional historical evidence of human audiovestibular disturbance following unquantifiable exposure. While several theories, such as the cavitation theory, have been postulated as a cause for symptomology, there is extremely limited knowledge of the pathophysiology behind the adverse effects that particular exposure frequencies, intensities, and durations have on animals and humans. This has created a knowledge gap in which much of our understanding is derived from retrospective examination of patients who develop symptoms after postulated exposures.

**Conclusion and Relevance:** Evidence for adverse human audiovestibular symptomology following exposure to acoustic waves and electromagnetic energy outside the spectrum of human hearing is largely rooted in case series or small cohort studies. Further research on the pathogenesis of audiovestibular dysfunction following acoustic exposure to these frequencies is critical to understand reported symptoms.

## Introduction

**Figure 1:**
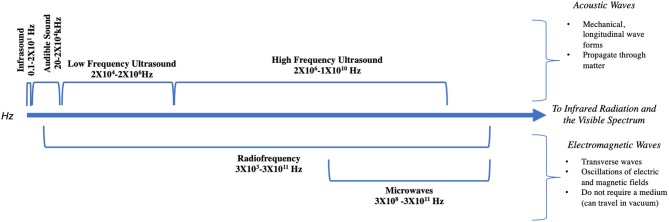
The frequencies of relevant acoustic and electromagnetic waves.

Acoustic and electromagnetic waves arise from mechanical vibrations of matter or the change in motion of charged particles, respectively, resulting in a transfer of energy (1). These two types of waves, while fundamentally different, oscillate at varying frequencies, measured in cycles per second, or hertz (Hz) (1) (Figure 1). Audiovestibular dysfunction following exposure to both acoustic and electromagnetic waves has been described as early as the 1910s (2), and has since been noted in all sectors, including industry and military. Throughout the twentieth century, occupation-related exposure to high-intensity acoustic and electromagnetic waves resulted in increasing awareness of symptoms such as dizziness, otalgia, hyperacusis, and hearing loss (3–6). As technological advancement expanded the ability to harness these forms of energy, increased daily exposure to these waves spurred additional reports (7, 8). Recently, the notion of an “acquired neurosensory disorder” gained heightened attention in the scientific (9, 10) and lay press (11) after reports emerged of individuals developing persistent neurological and audiovestibular symptoms after an exposure in Cuba (9).

In this review, we aim to examine the existing literature on adverse animal and human audiovestibular effects caused by exposure to wavelengths of acoustic and electromagnetic energy outside the audible range of sound (20–20,000 Hz). Given that many of these papers are retrospective case studies and occupation-related reports of audiovestibular dysfunction following unquantifiable and often undiscernible types of acoustic or electromagnetic wave exposure, there are inherent limitations to the conclusions made and the generalizability of the presented data. Additionally, in citing animal studies, we caution that while these models provide a tool to analyze exposure-related injuries, application to human injuries is limited. While we attempt to bring to the reader's attention any inconsistencies or major limitations with the referenced studies, our primary goal is to be comprehensive and inclusive in our survey of the published literature. In doing so, we hope to highlight the existing knowledge gaps regarding audiovestibular dysfunction following exposure to these wave forms, which may have significant implications for diagnosis, treatment, and prevention.

## Methods

A review of the literature was conducted using PubMed, Embase, and Scopus to identify articles related to adverse symptoms following exposure to sound or electromagnetic waves. Articles must have been written in the English language, and were identified by searching the MeSH terms “infrasound,” “audible sound,” “ultrasound,” “radiofrequency,” “microwaves,” “magnetic resonance imaging,” and “electromagnetic waves,” with combinations of “adverse health effects,” “clinical symptoms following exposure,” “auditory symptoms,” “hearing loss,” “tinnitus,” “hyperacusis,” “otalgia,” “vestibular symptoms,” and “dizziness.” Titles and abstracts were reviewed for potentially relevant articles, and full publications were obtained following this initial selection. The references of these articles were then reviewed in order to identify primary sources and literature not captured by electronic search databases. Additionally, auditory neuroscientists and clinicians treating patients with complaints following exposure were contacted to ensure inclusion of the most contemporary data and interpretations.

### Infrasound (0.1–20 Hz)

#### Infrasound Characteristics and Perception by Humans

Infrasound refers to the range of frequencies between 0.1 and 20 Hz (12). At high intensities, e.g., >100 dB sound pressure level (SPL), infrasound can be perceived by humans (12, 13). Infrasound is associated often with acts of nature, such as earthquakes, avalanches, or volcanoes. The earliest research on infrasound by Yeowart et al. and Von Bekesy examined whether infrasound could be perceived by humans (13–15). When heard, it is described as a “chugging, rough, popping, or motorboating” sound (16, 17). Due to its low frequency, infrasound penetrates walls, and other sound barriers with less attenuation than for higher frequency acoustic stimuli (18). Standard headband earmuffs, for example, tend not to attenuate infrasound to the ear, while earplugs attenuate infrasound better (16). While hearing sensitivity in humans generally declines for higher frequencies with increasing age, hearing sensitivity at these lower frequencies remains stable (13). Van den Berg showed that people between the ages of 50 and 60 years maintain hearing sensitivity in infrasound frequencies, with hearing thresholds only 4–5 dB HL lower than the median young person (19). This is noteworthy as many subjective complaints related to infrasound come from this older age group (13).

While largely outside the scope of this review on audiovestibular dysfunction from acoustic exposures, it is important to note that there have also been several theories that propose infrasound is perceived via proprioceptive mechanisms, rather than by the auditory system (20, 21). The most predominant one, known as the resonance theory, proposes that certain interceptors and proprioceptors within human tissues are activated when exposed to resonance frequencies within the infrasound range. Some researchers even suggested that essential danger to health occurs when exposed to infrasound at 7 Hz, which coincides with the alpha rhythm of brain waves, but this was never proven (20). Another experiment demonstrated oxidative phosphorylation and increased membrane penetration in erythrocyte intracellular membranes *in vitro* following infrasound exposure, but the primary cause remains unknown (20). Additionally, a more recent theory based on rat studies proposes that a period of infrasound exposure may induce apoptosis and upregulate calcium concentrations in hippocampal neurons, suggesting damage to the Central Nervous System (22).

#### Audiovestibular Symptoms Following Infrasound Exposure

Researchers began investigating auditory and non-auditory effects of infrasound exposure following sensational reports by Gavreau et al. in the mid 1960s (23). Gavreau stated that “weak infrasound could affect the balance or equilibrium mechanism in the ear, produce fatigue, induce nausea, etc.” (16). He attributed modern-day “city fatigue” to infrasound (17), and said infrasound was “certainly one of the many causes of allergies, nervous breakdowns, and other ‘unpleasant phenomena of modern life’ found in industrial cities.” Other scientists at the time disagreed, noting that Gavreau's claims lacked any scientific basis (17); nevertheless, the statements sparked newspaper speculation about infrasound ray guns, the ability for infrasound to make drivers act inebriated, or even as a cause of brain tumors (17).

Following the Gavreau et al. article, the subsequent primary literature following infrasound exposure describe infrasound as causing complaints, specifically “annoyance” and “unpleasantness.” One of the first reports was described by Bryan et al., who reported on two residents living near a factory boiler that complained of auditory annoyance even though the infrasound decibel level outside their house was only 55 dB SPL (24). Importantly, the authors were unable to rule out the possibility that these effects arose from other spectral components that may have also been emitted by the factory boiler. Other sources of infrasound annoyance have been reported in relation to the Concorde engine test bed, air-conditioning systems, and oil-fired burners and boilers (25). In another case study, complaints about low frequency noise in Southern England were followed by noise measurements. People exposed to this sound described a “throbbing” sensation, which was more prominent indoors than outside (25). The authors note that decibel level was not a good predictor of reported annoyance, which is corroborated by other studies (17, 25).

Several experiments attempted to examine quantitatively auditory consequences following infrasound exposure. The threshold for aural pain at infrasound frequencies is 140 dB SPL at 20 Hz, 162 dB SPL at 2 Hz, and 175–180 dB SPL for static pressure (26). Von Gierke and Nixon noted that eardrum rupture occurs at 185–190 dB SPL (26). While multiple factors affected the ability to perceive infrasound audibly, the sound pressure level had the greatest effect (12). These studies also demonstrated that in order to elicit human perception of infrasound, higher sound pressure levels are necessary at lower frequencies. Additionally, the duration of exposure to the stimulus and the frequency of infrasound play a role in determining how the sound is perceived. Experiments modifying these characteristics were used to determine audible and pain thresholds from infrasound (12, 26, 27). Tonndorf was the first to report otologic damage following long-term exposure to infrasound (28). He described scarring of the tympanic membrane in German submariners exposed to diesel rooms of submarines and presumed the damage was due to exposure to infrasound, though the duration, intensity and frequency of exposure to infrasound was unknown (17, 28). Additionally, a major limitation of this study was the inability for the authors to separate out other higher frequencies in the noise spectrum that submariners could have possibly been exposed to.

In preparation for both the Apollo program and exposure to high levels of infrasound from the Saturn V rocket, Mohr et al. exposed subjects to 140–150 dB SPL of infrasound (17, 27). It was concluded that with ear plugs, astronauts self-reported being able to tolerate 2 min of exposure after takeoff, which is when the sound would be most intense. Mohr also noted performance decrements during pointer following tasks at high decibels of low frequency noise (13, 27, 29). Additionally, aural fullness and pain after infrasound exposure has been cited in multiple studies, with symptoms occurring at infrasound intensities beginning at 127–133 dB SPL but do not become more intense as sound pressure increases (17, 27, 30–35).

The multiple studies exploring vestibular consequences from infrasound exposure reported inconsistent results. Two studies described the presence of vertical nystagmus, while many reported the absence of nystagmus (17, 18, 31, 36–41). Other studies have found a minor vestibular effect at 110–120 dB SPL, while others have found no effect (5, 16–18, 42). Bruel and Oleson reported that they were able to produce dizziness in subjects exposed to a level of 95 dB SPL at 2 Hz over the course of 2 h, and this is the only published report of dizziness with infrasound exposure (17, 43). Broner notes that infrasound becomes a vestibular hazard to humans at least above 130 dB of sound pressure (17, 18, 33).

#### Wind Turbine Syndrome

In the past two decades, the bulk of infrasound research has focused on “wind turbine syndrome,” the effect of low frequency sounds from wind turbines that have been reported to cause sleep disturbance, headaches, difficulty concentrating, irritability, fatigue, dizziness, tinnitus, and aural pain (44–50). This phenomenon is not fully understood, and ongoing research continues to study how low-level infrasound may be causing vestibular consequences (51–56).

Salt et al. propose that infrasound may affect different components of the ear without producing audible sound. Specifically, he explains that A-weighting, a commonly used filter function applied to instrument-measured sound levels in order to correct for the relative perceived loudness of that sound by the human ear, corresponds to inner hair cell responses in the ear. However, because infrasound has been shown to stimulate outer hair cells without any inner hair cell response and subsequent perceived hearing, using A-weighting to measure the frequency spectrum of sound generated by wind turbines is fundamentally flawed (49). Without the use of A-weighting, Van den berg demonstrated that at 1 Hz, the sound pressure level of wind turbines approached 90 dB SPL, and Sugimoto et al. showed that at 2 Hz, sound pressure of wind turbines approached 100 dB SPL (19, 49, 57, 58). Salt and colleagues conclude that infrasound may be affecting peripheral auditory and vestibular structures through mechanisms that avoid audible detection (49).

### Audible Sound (20–20,000 Hz)

#### Human Perception of Audible Sound

While an in depth discussion of acoustic trauma in the audible sound range is beyond the scope of this article, numerous studies have demonstrated auditory damage following high intensity environmental noise exposure (59–63) (Table 1). Additionally, one of the first major investigations of the biological effects of “noise” on humans was published as the BENOX report in the 1950s, but the authors could not clearly delineate what type of acoustic or electromagnetic exposures may be contributing to the effects they outline (64). Finally, the neurobehavioral effects of high intensity audible sound also have been used as a tool for crowd control. There are a few notable examples of the use of audible sound as deterrents that are worth highlighting.

**Table 1:** Current noise exposure guidelines^a^.

**Time to reach 100% noise dose**	**Exposure level** **(NIOSH^**b**^) (dBA)**	**Exposure level** **(OSHA^**c**^) (dBA)**
15 min	100	115
30 min	97	110
1 h	94	105
2 h	91	100
4 h	88	95
8 h	85	90

a *Adapted from CDC (CDC 2018)*.

b *The National Institute of Occupational Safety and Health*.

c *Occupational Safety and Health Administration*.

#### Audible Sound as a Human Deterrent

The long-range acoustic device (LRAD) is among the best described examples of sound deterrents. Designed by the LRAD Corporation, LRADs are denoted as hailing and warning devices (65). It is a flat, loudspeaker system which can be targeted in a certain direction, transmitting sound waves within the audible range of frequencies. There are two settings, a warning mode that can release 151 dB SPL over 1,000 m distance, and a voice mode that can release 121 dB SPL over 500 m, both of which can emit at frequencies between 1 and 10 kHz. Ear plugs and earmuffs can provide 15–45 dB SPL of attenuation, although annoyance and distraction has still been shown to occur (65). A newer LRAD model, known as the LRAD 200X, is being advertised as having effectiveness for distances up to 5,500 m (66). Permanent ear damage and otalgia can occur when individuals are within a range closer than that for which the system was designed (65). To our knowledge, there are no published, publicly available scientific studies examining auditory or vestibular effects from exposure to the LRAD.

Another example of noise used as a human deterrent is the Mosquito, designed by a British company called Compound Security Systems. The Mosquito is a sonic repellant that was designed to repel young people, who are capable of hearing its emitted sound between 18 and 20 kHz. As people age, they lose hearing at the highest frequencies of the audible range, such that only younger people can hear the frequencies emitted by the Mosquito (67). Anecdotal testimonials describe its ability to deter and disperse teenagers (67). Similar to the LRAD, no scientific studies on its effects have been conducted to date.

Additional examples include SoundLazer speakers used in museums to project sound in a local field while not disturbing neighboring areas.

### Low Frequency Ultrasound (17.8 kHz−2 mHz)

#### Ultrasound Wave Characteristics

Historically, ultrasound has been defined as sound waves with frequencies greater than what is audible to most humans (6). Today, the scientific community defines ultrasound more precisely as sound waves that exceed 17.8 kHz (8, 68). Because of ultrasound's short wavelength (17 mm at 20 kHz in air), when traveling through air it is less susceptible to diffraction due to environmental objects or local atmospheric conditions compared to audible sound (6). Additionally, the velocity of ultrasound through air is the same as audible sound (343 m/s), but it is readily attenuated by air and cannot be transmitted over long distances, even when beamed and focused (6). Critical to studying the effects of ultrasound on biological tissues is understanding the amount of energy that can be transmitted across media, such as from air to human skin. Compared to audible sound, an even greater impedance mismatch occurs when sound waves travel from media with significantly different densities, resulting in a reflection of these waves rather than transmission (69). For example, only about 0.1% of airborne ultrasound energy is transmitted from air to human tissue, as the air/tissue interface provides a highly reflective boundary (6, 69). The mammalian middle ear, however, has evolved to improve impedance matching for audible sound between air and the fluid-filled inner ear, and also can transmit ultrasound to the inner ear.

#### Historical Evidence of Audiovestibular Dysfunction Following Ultrasound Exposure

The body of research on audiovestibular dysfunction from ultrasound waves emanated from two major historical eras: the industrial use of ultrasound beginning in the mid twentieth century and the recent use of ultrasound in commercialized devices. Adverse audiovestibular symptoms related to ultrasonic noise exposure was first described in the mid 1940s in aircraft personnel who worked frequently with jet engines (3, 7, 8, 70–72). A constellation of symptoms including fatigue, headache, nausea, vomiting, unsteadiness, temporary tinnitus, and aural fullness was coined “ultrasound sickness” (3, 6, 70). Similar to Tonndorf and Broner's study on submariners, a major limitation of these conclusions was the inability for scientists to decipher exactly what frequencies these aircraft personnel were exposed to. This significant flaw in the study design limits its conclusions.

The first legal case for injury by ultrasound occurred in 1948 in England, after which the US Aeronautical Board formed the Ultrasonics Panel to investigate the biological effects of ultrasound (7, 70). In 1951, Dickson and Chadwick proposed that these symptoms were caused by vestibular disturbance from intense spectral components in the audible frequency range (73). However, Dickson and Chadwick's report was also met with considerable scrutiny. Davis and his colleagues published a report in 1949 which posited that there was no evidence that ultrasound exposure was a hazard to hearing (70). Additionally, Parrack argued that ultrasound sickness appeared to be psychosomatic in origin, spurred largely by the media's sensationalization of the phenomenon and subsequent public apprehension (3), although not well-substantiated.

In 1955, Crawford published a novel case series linking ultrasound exposure to disequilibrium, fatigue, nausea, and headaches in laboratory workers from the UK that persisted after the exposure ended, as well as “loss of hearing in the upper audible frequencies” (74). Several years later, Skillern correlated similar symptoms with a frequency band of ultrasound centered at 25 kHz, and gave one of the first descriptions of ultrasound-associated ear pain from ultrasound devices for cleaning, welding, and drilling, stating “this pain was similar to a burning sensation in the auditory canal… pain was experienced more rapidly than while measuring other devices with sound pressure levels of greater intensity” (75). Parrack published an additional study focused specifically on hearing changes after ultrasound exposure, and found temporary threshold shifts at subharmonics as a result of 5-min exposure to discrete ultrasound frequencies between 17 and 37 KHz at 48–143 dB SPL, which resolved almost immediately after exposure. This evidence bolstered his assertion that ultrasound did not cause any permanent auditory damage (7, 76). However, it is important to note that this notion has been discredited, as recent data suggests that repeated temporary thresholds shifts results in permanent synaptopathic injury that may not be registered by conventional audiometric evaluation, termed “hidden hearing loss” (62, 77).

One of the most robust investigations of industry-related ultrasound exposure was completed by Acton and Carson in 1966, who examined the intensity and frequency of ultrasound-emitting equipment such as ultrasonic washers and drills and performed audiometric tests on exposed personnel prior to and after acute exposure (78). They noted that ultrasonic cleaning baths, which produced 95 dB SPL at 20 kHz and 115 dB SPL at 40 kHz, caused complaints of fatigue, buzzing, nausea, and headaches in workers, symptoms which could last for several hours after exposure ceased. Interestingly, all of these effects disappeared once the machines were placed in an enclosure. They also investigated ultrasound exposure from drills, and surprisingly found that none of the workers reported any audiovestibular symptoms. However, it is unclear what type of reporting biases that may have affected their responses, such as potential fear of losing their positions. The authors themselves complained of persistent ringing and aural fullness from brief exposure to the noise from the drills at the time of the study. Like Parrack, Acton and Carson did not find any permanent threshold shifts in hearing, although their studies did not test hearing above 12 kHz.

Since Acton and Carson's study, several other studies of ultrasound-related audiovestibular symptoms from industrial machines were published (6, 23, 26, 72, 79, 80). Mixed conclusions regarding permanent audiovestibular damage as a result of ultrasound exposure were reached, and many argued the evidence base across all occupation-related studies were not generalizable to the general public (7). Another confounding variable was the difficulty in separating the effects of ultrasound from the high intensity audible noise that often accompanied industrial ultrasonic machines. One early study conducted by Ades et al. attributed symptoms of ultrasound sickness to high levels of sound in the audible frequencies rather than ultrasound (64). An additional study several decades later found that all ultrasound emissions from a welding factory were accompanied by subharmonics in the audible high-frequency range as “a byproduct of industrial ultrasonic processes,” making it difficult to parse out whether the audiovestibular symptoms that industrial workers reported were related to ultrasound or high intensity audible noise (81).

#### Ultrasound Exposure From Consumer Devices

Recent research of environmental ultrasound exposure has transitioned from industrial workers to everyday consumers. Newer technology has also enabled ultrasound production without accompanying audio frequency emissions (7). These ultrasonic sources include devices such as wireless phone chargers, TV remote controls, automated door openers, humidifiers/vaporizers, and pest repellents. In a 2016 review, Leighton et al. suggested that the most common source of high intensity airborne ultrasound in today's public spaces are on public address (PA) systems that are found in stadiums, schools, workplaces, and public transport vehicles (7). Most of these device manufactures do not report the frequencies or intensities their products produce, but argue they are 50 times lower than the lowest ultrasound imaging exposure limits set by the FDA for medical imaging (7). However, it is important to note that the mechanism by which ultrasound is transmitted in medical imaging is through direct skin contact, which is a different mechanism than what is discussed in this review.

In a 2014 study by Ueda and colleagues, patrons at a restaurant were exposed to an acoustic insect repellent that produced 20 kHz ultrasound waves between 90 and 130 dB SPL (82). They noted that only those patrons who heard the source reported ear pain, discomfort, or irritation, supporting a theory raised by Leighton that US exposure may more adversely affect those that can perceive high frequency sound (7). Another study by Glorieux and Van Wieringen used more controlled conditions and exposed participants to both audible and inaudible levels of ultrasound for 20 minutes (83, 84). They found little to no symptomology in their subjects, however the intensity of the sound was low (45–70 dB SPL). Lastly, a recent study by Fletcher et al. sought to delineate how very high frequency sound or ultrasound (VHFS/US) may cause adverse physiological and psychological symptoms in a cohort who had a history of auditory complaints from US exposure in public vs. an asymptomatic cohort (68). They found that both cohorts exposed to 20 kHz (82–92 dB SPL) had a statistically significant difference in discomfort with VHFS/US exposure compared to a 1 kHz reference stimulus, but this was meaningfully different only in the symptomatic group (mean increase of 1.9 points on an 11-point scale compared to 0.3 points in the asymptomatic group). The symptomatic group also reported meaningfully increased annoyance and difficulty concentrating. The authors found no significant difference for either group in performance on a sustained attention task after VHFS/US exposure compared to 1 kHz exposure.

#### Proposed Mechanisms for Audiovestibular Disturbance

Leighton argued that there is a paucity of data on the frequency of VHFS/US exposures and what, if any, audiovestibular symptoms are linked to these technologies (7, 8). He also highlighted the lack of scientific evidence behind a mechanism for audiovestibular disturbances from ultrasonic waves. One proposed mechanism, “acoustic cavitation” is a biophysical phenomenon following ultrasound exposure, in which an ultrasonic wave traveling through a liquid can cause the growth, oscillation, and collapse of bubbles (69, 85–87). As a consequence of the bubble's collapse, energy is released, resulting in acoustic wave emission (86). The effects of cavitation can manifest within the bloodstream, or in other fluids such as cerebrospinal fluid, endolymph and perilymphatic fluid, causing disturbances in audiovestibular physiology; however, the damage of the collapsing of cavitation bubbles on local tissue remains largely unstudied (86). Additionally, in his 2016 review, Leighton offers another untested hypothesis, stating that with “intense ultrasound,” the tympanic membrane undergoes microscopic displacement, activating tympanic membrane proprioceptors and middle ear and Eustachian tube muscles. Another mechanism for audiovestibular disturbances from ultrasound waves proposes that vestibular otolith organs are activated through acoustic radiation force (88).

### Radiofrequency (3 kHz−300 GHz) and Microwaves (1–30 GHz)

#### Electromagnetic Wave Properties

The properties of electromagnetic waves are fundamentally distinct from sound waves in many ways (Figure 1). While sound waves are mechanical vibrations that propagate through matter and carry energy, electromagnetism does not require a medium and can travel through a vacuum (1). Electromagnetism is described as being both waves and photon particles (the wave-particle duality of quantum mechanics). However, like sound waves, electromagnetic waves have an oscillating frequency and can be depicted along a spectrum beginning with radio waves at the lower end and Gamma rays at the upper end (89). Indeed, acoustic and electromagnetic waves are related; researchers have recently discovered that it is possible to transduce energy from sound waves into electromagnetic waves (90). Electromagnetic waves have also been found to have auditory precepts on animals and humans (91–93), and are therefore within the purview of this paper.

#### Radiofrequency Hearing and the “Frey Effect”

The phenomenon of hearing radiofrequency energy is called radiofrequency (RF) hearing or the microwave auditory effect (94). The terms “microwave” and “radiofrequency” are often used interchangeably in the literature, even by the bodies that regulate RF exposure (95). The Federal Communications Commission (FCC) defines RF energy as a category within the electromagnetic spectrum between 3 kHz and 300 GHz (96). Microwaves are defined by the FCC as the frequency band between 1 GHz and 30 GHz. Emitters of RF energy include computers, television, radio antennas, microwave ovens, police radars, ablative surgery, and MRI. The first report of auditory percepts from RF energy was in 1947 at the Airborne Instruments Laboratory in Mineola, NY. Standing close to a 75-foot-tall antenna, several people felt that “sound was produced in the head without any direct acoustic input.” A description of the experience was published in 1957 and served as the catalyst for research on RF energy and its acoustic properties (97).

The phenomenon of hearing RF energy was described further in multiple experiments by Allan Frey and is commonly referred to as the Frey Effect or the “microwave auditory effect” (MAE). Prior to Frey's first review in 1961, there was no scientific report of hearing electromagnetic energy. After reproducing the audible effect of RF energy, Frey's subjects described the sound as a “click, buzz, hiss, knock, or chirp” (98–100). Through various experiments on humans, Frey reported that the ability to hear air-conducted sound in the range of 5–8 kHz is a requirement for hearing RF energy (98, 101, 102). Additionally, hearing RF energy was contingent upon a quiet environment (98–100, 103, 104), as outdoor experiments demonstrated normal ambient noise levels masked the ability to hear RF energy (103).

Initially, the mechanism behind RF hearing was thought to involve direct stimulation of neurons (99). However, Frey and colleagues showed that RF energy stimulated the cochlea, producing cochlear microphonics (electrical potentials), in a manner similar to acoustic stimuli (92, 105). The cochlea was later confirmed to be the transducer of RF energy by experiments in both humans and cats, in which destruction of the cochlea eliminated RF hearing (103, 106–108).

Eventually, a pathway to describe RF hearing was proposed after a series of theoretical, animal, and human studies. Known as the thermoelastic expansion theory, this idea suggests that upon absorption of RF energy, there is a small and quick rise in temperature (~10^−6^°C), which expands tissue, and launches an acoustic pressure wave, which is perceived in the cochlea, and then relayed to the central auditory system (106, 109, 110). The pulse of energy can be brief (<50 ms), but the peak power of the pulse must be strong enough to induce RF hearing (500–5,000 mW/cm^2^) (106, 109). Since this theory was originally proposed, additional experiments have been conducted to further characterize the ways in which RF hearing can be augmented, such as by increasing the microwave pulse width (100, 111).

#### Historical Data on RF Exposure From Eastern Europe in the Mid 1900s

The first reports of adverse health consequences resulting from RF energy came from research conducted in the Soviet Union and Eastern Europe between 1933 and 1965 (4). The most frequently reported human responses to RF energy in the Soviet literature were noted for frequencies of 30–300,000 MHz at both thermogenic and non-thermogenic intensities. Auditory and vestibular symptoms reported in the Soviet and Eastern European literature included pain in the head and eyes, weakness, weariness, and dizziness, and headache (4). This research informed Soviet and Eastern European standards for maximal permissible exposure during the workday.

In a review of available Soviet and Eastern European literature examining the clinical manifestations following microwave exposure, Dodge reported that there is a paucity of data on the circumstances of irradiation, frequencies used, effective area of irradiation, orientation of the body with respect to the energy source, waveform (continuous or pulsed), or exposure schedule or duration. In many Soviet scientific papers, multiple experiments are cited, but adverse symptoms are discussed generally and are not linked with individual experiments or conditions. Moreover, many of these experiments have not been replicated (4).

#### Animal and Human Studies Following RF Exposure Produced Varied Symptoms

Among western scientific literature, there are only case reports and retrospective case series suggesting adverse health effects from RF exposure in humans (112–115). Guy and Chou recorded the first instance of adverse effects on rats (116). After delivering RF pulses of varying width and peak power to the rats, the authors observed neuronal demyelination after 1 day, and brain swelling at 1 month. Importantly, however, the stimulus produced an absorbed energy of 680 J, or 28 kJ/kg, which is approximately 100,000 × higher than thresholds for auditory responses in humans (116). Soon after, Wachtel et al. and D'Andrea et al. studied the effectiveness of high peak power microwave pulses on reflexive movements in mice, finding that bursts at 1,250 MHz produced “agitation movements” (117–119).

Beginning in 1980, several case reports on isolated incidents of adverse health effects from human exposure to high-intensity RF energy were published (112–115). Many of these reports focused on exposure to RF energy from antennas. Williams and Webb reported two airmen exposed to RF radiation 38 × greater than Air Force permissible levels had suffered from anxiety and hypertension (112). In 1982, Forman et al. reported on two men accidentally irradiated with microwave RF who developed emotional lability, irritability, headaches, and insomnia (113). Schilling was the first to describe audiovestibular symptoms in this population after detailing six antenna engineers in two separate incidents exposed to 100 MHz RF radiation. They experienced headaches, malaise, dizziness, and ear pain (115).

#### Historical Evidence of Human Audiovestibular Disturbance Following Exposure

One interesting report of adverse symptoms from RF energy occurred between 1953 and 1976, when microwave beams were focused on the American embassy in Moscow. The US State Department commissioned a report, the Johns Hopkins Foreign Service Health Status Study, published in 1978 and commonly known as the Lilienfeld study after the report's author, that contains medical data and exposure parameters (120). From 1953 to 1975, a maximum of 5 μW/cm^2^ at a frequency between 2.5 and 4.0 GHz were beamed and focused on the upper half of the Embassy's Chancery building for 9 h/day. From June of 1975 to February of 1976, a maximum of 15 μW/cm^2^ at a frequency between 2.0 and 3.0 GHz were focused on a different part of the building for 18 h/day. The RF beam from 1953 to 1975 originated from an apartment building 100 m west of the Chancery building and from 1975 to 1976 from two sources, buildings 100 m to the east and south. Over 1,800 employees were identified as having been exposed and their symptoms were compared to their colleagues in nine Eastern European embassies. There was no evidence of increased mortality in the Moscow group compared to people stationed at other embassies. However, hundreds of analyses were also conducted in order to determine differences in non-fatal comorbidities, and Lilienfeld et al. noted an increased frequency of depression, irritability, difficulty concentrating, and memory loss in those working in the US Embassy in Moscow (120, 121). It is important to note that the author found no direct relationship between these symptoms and microwaves, and the strongest differences between the Moscow group and the comparison group came from people who had the lowest exposures determined by number of days at the Embassy. The irradiation of the US embassy in Moscow is the only recorded experience of chronic, long-term human exposure to microwave energy. Lilienfeld et al. acknowledged limitations of this unique paper including low response rates to health survey questionnaires, poor and/or incomplete health records for many individuals, and difficulty classifying the amount of microwave exposure (121).

Later articles reviewing these findings question the author's limited conclusions, and attempt to provide additional evidence to support the notion of “RF sickness syndrome” (122). Liakouris notes that the Lilienfeld study does not provide an explanation for certain findings in his conclusions. These include autoimmune diseases, neurological problems (“diseases of the peripheral nerves and ganglia among males”), reproductive problems (problems during childbirth, pregnancy, and puerperium) and tumors. She also notes that two functional deficits—concentration difficulties and refractive eye problems—are unaccounted for in the Lilienfeld study conclusions (123). Goldsmith states that the Lilienfeld study assumes that the US embassies in other Eastern European countries were not exposed to RF radiation. Additionally, he explains that there is documented evidence of “downgraded” concerns amongst the Johns Hopkins team following a conference with the United States State Department (124).

Though evidence and research on radiofrequency disturbance is pervasive, we acknowledge biases in the field of auditory research has led to a more robust radiofrequency research base, and we caution readers not to draw conclusions based on the size of research volume.

### Magnetic Resonance Imaging

#### Isolated Reports of Auditory Symptoms Following Clinical MRI Exposure

Magnetic resonance imaging (MRI), which utilizes pulsed radiofrequency waves to generate images, has also raised concerns within the scientific community for its potential harmful effects on audiovestibular functioning (125–127). MRI uses a powerful magnet, usually 1.5 or 3T in clinical MR settings, to cause proton alignment within exposed human tissue. When additional energy in the form of a radio wave is added, the magnetic field vector is temporarily deflected, and its return to baseline causes a RF signal to be emitted, which is used to generate the MR images (128). Large magnetic gradient coils and varying lengths of RF pulse sequences alter the strength and characteristic of the magnetic field and resultant RF signals it produces in order to isolate different slices of body tissue of interest to the radiologist (128, 129). In a computational study evaluating the intensity and frequency of thermoelastic pressure waves generated by RF pulses specifically from 1.5, 7, and 9T MRI scanners, Lin and Wang found that even at specific absorption rates (SARs) much higher than the FDA guideline of 8 W/kg in a clinical setting, the sound pressure levels produced by RF pulses would not be more than 17 dB above threshold, suggesting that auditory trauma would be unlikely from RF waves alone (130).

Along with the studied RF wave effects on human audiovestibular dysfunction, acoustic noise from the magnetic field generated from an MR machine is also a consideration for potential acoustic trauma. A Lorentz, or electrical, force acts on MR gradient coils as the gradient current switches direction and magnitude. This force vibrates the coil mountings, leading to the emission of audible sound waves, which have been inconsistently reported in the literature to cause tinnitus, hyperacusis, and permanent hearing damage (125–127). However, several studies have only demonstrated a temporary auditory threshold shift immediately after an MRI scan which resolved in follow-up, as well as clinically non-significant long-term auditory thresholds when earplugs were used during MR exposure, although the authors mention that the data on this subject is limited (127, 131, 132).

#### Vestibular Disturbances Following MRI Exposure in Animal Models and Humans

While auditory findings from prolonged MRI exposure have been limited to isolated case reports, vestibular dysfunction from this technology has been more thoroughly examined in animal and human experiments. Vertigo and nausea were initially reported in an early human study on exposure to a 4T MRI magnet, and more recent reports have described transient dizziness and vertigo in patients, research subjects, and health care workers exposed to MRI scanners (133–136). Additionally, upon exposing rodents to 7T, 9.4T, and 14.1T static magnetic fields, researchers observed locomotor activity, conditioned taste aversion to glucose-saccharine and c-Fos activation, an immediate-early gene activated during neural activity, in visceral and vestibular nuceli (137, 138) which the authors noted was similar to responses from vestibular stimulation, suggesting that magnetic field exposure may produce a vestibular disturbance (131). While several mechanisms had been proposed to explain the relationship between dizziness and MRI machines, in a 2009 functional MRI (fMRI) study on the caloric test's stimulus on the brain, Marcelli incidentally noted nystagmus in patients who were inside a 1.5T MRI machine before the caloric stimulation even began (139, 140). Subsequently, Roberts et al. systematically studied nystagmus in humans exposed to 7T MRI and proposed that a Lorentz force occurs in the labyrinth of healthy humans in an MRI, generated by the interactions of the strong static magnetic field and natural electric currents entering hair cells of the utricle (141). To date, this hypothesis explains the vertigo and nystagmus experienced by humans, rats and mice in magnetic fields (142). Importantly, this effect occurred due to the static magnetic field alone and not from radiofrequency pulses or time-varying magnetic fields. Our research did not uncover any reports of non-clinical MRI use, perhaps due to weight and size factor considerations in deployment of an MRI.

## Limitations and Conclusions

Scientific advancements in the use of different frequencies of acoustic and electromagnetic energy have raised the specter for audiovestibular dysfunction following exposure. However, scientific evidence for adverse human audiovestibular symptoms following exposure to acoustic and electromagnetic waves is largely rooted in small case series or cohort studies, with limited conclusions. Thus far, much of our understanding is derived from retrospective examination of symptomatic patients who self-report exposure to a form of acoustic or electromagnetic energy. Given this, there exists inherent difficulty in making connections between the type, duration, amount of exposure, and subsequent injury. Ideally, scientists would attempt to delineate how “chronic” vs. “acute” exposure may impact the human cochleovestibular system and manifest in clinical symptomology, as well as determine an inflection point between transient to permanent damage. However, the scarcity of human *a priori* research makes this task difficult.

While largely outside the scope of this review, pharmaceuticals have shown efficacy in animal models to mitigate noise-induced hearing loss (NIHL), and may play a role in the advancement of therapies for neurosensory dysfunction. One paper investigated the bio-availability of various anti-oxidants in cochlear fluids in an animal model (143). An additional study by Grondin investigated the susceptibility of individuals to NIHL based on genetic and environmental factors (144). However, it is important to note that animal models are mono-morphic, while humans are poly-morphic, offering multiple metabolic pathways to intercept hearing loss. Nevertheless, pharmaceutical efficacy is a complex subject which shows some promise in growing the body of literature within neurosensory dysfunction.

Our research aim was to examine adverse audiovestibular consequences following energy exposure, but we acknowledge that further research is necessary to examine the effects of energy exposure to organs outside the audiovestibular apparatus. Additionally, we acknowledge that future studies may further evaluate the merits of the cited papers. Finally, further research in the public sector on the pathogenesis of audiovestibular dysfunction following exposure to these stimuli is critical to better understand the health effects on humans and the potential role of therapeutic intervention to mitigate adverse effects.

## Author Contributions

RL, NK, RK, AR, and EK contributed conception and design of the study. RL and NK wrote the first draft of the manuscript. All authors contributed to manuscript revision, read and approved the submitted version.

## Conflict of Interest

The authors declare that the research was conducted in the absence of any commercial or financial relationships that could be construed as a potential conflict of interest.
